# Thyroid Dysfunction and Dysmetabolic Syndrome: The Need for Enhanced Thyrovigilance Strategies

**DOI:** 10.1155/2021/9641846

**Published:** 2021-03-29

**Authors:** Sanjay Kalra, Sameer Aggarwal, Deepak Khandelwal

**Affiliations:** ^1^Bharti Hospital and B.R.I.D.E., Karnal, India; ^2^Apex Plus Super Speciality Hospital, Rohtak, India; ^3^Khandelwal Diabetes, Thyroid & Endocrinology Clinic, Delhi, India

## Abstract

Thyroid dysfunction (TD) is common in metabolic disorders such as diabetes mellitus (DM), cardiovascular disease (CVD), obesity, dyslipidemia, hyperuricemia, kidney and liver dysfunctions, and polycystic ovary syndrome (PCOS). Subclinical hypothyroidism (SHypo) worsens glycemic control in patients with DM, and these patients, especially those with Type-1DM, have higher prevalence of TD. Both TD and DM increase CVD risk. Even minor alteration in thyroid hormone (TH) levels can alter cardiovascular function. While hyperthyroidism increases systolic blood pressure and leads to high-output heart failure, hypothyroidism increases diastolic blood pressure and leads to low-output heart failure. Chronic subclinical hyperthyroidism (SHyper) and SHypo both increase the risk of hypertension, coronary artery disease (CAD) events, CAD deaths, and total deaths. SHyper alters cardiac morphology and function. SHypo causes dyslipidemia and endothelial dysfunction and increases the risk for weight gain and obesity. Overweight and obese patients often have hyperleptinemia, which increases the secretion of thyroid stimulating hormone (TSH) and induces TD. Dyslipidemia associated with TD can increase serum uric acid levels. Hyperuricemia promotes inflammation and may increase the risk for dyslipidemia, atherosclerosis, and CVD. TD increases the risk for developing chronic kidney disease. In nephrotic syndrome, proteinuria is associated with urinary loss of TH leading to TD. Some correlation between TD and severity of liver disease is also seen. TD and PCOS have common risk factors and pathophysiological abnormalities. Hypothyroidism must be excluded before diagnosing PCOS. Current guidelines do not strongly recommend thyroid screening in the presence of all metabolic disorders. However, pragmatic thyrovigilance is required. Clinicians must stay alert to signs and symptoms of TD, maintain high clinical suspicion, and investigate thoroughly. Drug-induced TD should be considered when TH levels do not match clinical findings or when patients are on medications that can alter thyroid function.

## 1. Introduction

Dysmetabolism is a frequently encountered comorbidity and complication of thyroid dysfunction (TD). TD is often seen as a comorbidity of dysmetabolism. TD may occur as overt or subclinical hypo- or hyperthyroidism. In overt hyperthyroidism, thyroxine (T4) or triiodothyronine (T3) levels are elevated and thyroid stimulating hormone (TSH) levels are <0.1 mIU/L or undetectable [1]. In overt hypothyroidism, T4 level is low, but TSH level is >4.5 mIU/L [1]. In subclinical hyperthyroidism (SHyper), T3 and T4 levels are normal, but TSH levels are <0.4 mIU/L [1]. In subclinical hypothyroidism (SHypo), T4 levels are normal, but TSH levels are 4.5 mIU/L [1]. This review summarizes current knowledge about TD and its comorbidities contributing to metabolic syndrome and discusses clinical implications for the management of these conditions that may require enhanced thyroid screening recommendations. We searched PubMed for any combination of *thyroid dysfunction*, *hyperthyroidism*, *thyrotoxicosis*, or *hypothyroidism* AND *metabolic syndrome*, *diabetes mellitus*, *glucose*, *blood pressure*, *hypertension*, *hyperlipidemia*, *cholesterol*, *body weight*, *hyperuricemia*, *liver*, *kidney*, or *polycystic ovarian syndrome*. We included papers and reviews published between 2010 and 2020. We also searched for current international guidelines mentioning thyroid screening.

## 2. Metabolic Dysfunctions

### 2.1. Glucometabolic Dysfunction

TD and glucometabolic disorders such as diabetes mellitus (DM) or prediabetes are the most frequent chronic endocrine disorders [2]. Both TD and DM add to the risk of cardiovascular disease (CVD).

#### 2.1.1. Dysglycemia in Thyroid Dysfunction

According to a study, low thyroid function, even in the low-normal range as seen in subclinical hypothyroidism (SHypo), increases the risk of diabetes by about 13% [3]. However, conversion of prediabetes to diabetes is higher (35% vs 15%) in those with low-normal thyroid function (SHypo) than in those with normal thyroid function [3].

Glucose metabolism is altered in both hypothyroidism and hyperthyroidism. Hypothyroidism is characterized by decreased glucose absorption, decreased hepatic glucose uptake, decreased or normal hepatic glucose output, and reduced liver and muscle gluconeogenesis and glycogenolysis. Hyperthyroidism, on the other hand, is associated with increased energy expenditure and weight loss despite increased appetite and food intake, reduced cholesterol levels, increased lipolysis, and gluconeogenesis [2].

#### 2.1.2. Thyroid Dysfunction in Diabetes

Prevalence of TD among patients with type 2 DM (T2DM) ranges from 9.9% to 48% [4]. TD is more common among patients with type 1DM (T1DM) than those with T2DM [4].

Among patients with T1DM, autoimmune thyroid disorder (AITD) is the most frequent autoimmune comorbidity that can manifest as autoimmune-induced hypothyroidism (Hashimoto's thyroiditis) or hyperthyroidism (Grave's disease) [5]. AITD is seen in 17%–30% of adults and in 25% of children with T1DM [2, 6–8]. Patients who are positive for thyroperoxidase antibodies (anti-TPO) are 18 times more likely to develop hypothyroidism [2]. AITD is more common among females with T1DM and in patients with longer duration or poorly controlled diabetes [2].

Both hypo- and hyperthyroidism are more prevalent among patients with T2DM than in the general population [4]. Hypothyroidism is prevalent in 6%–20% of patients with T2DM [2, 9–11] and is a risk factor for new onset DM, especially in those with prediabetes [3]. Hypothyroidism is considered to be a risk factor only in patients taking statins [12]. Hyperthyroidism worsens glycemic control in T2DM by affecting several different organs [4]. It can worsen subclinical DM and increase risk of diabetic complications in patients with T2DM. Conversely, poor glycemic control in elderly women with T2DM is also associated with increased risk of subclinical hypothyroidism that can lead to insulin resistance [13]. The resultant hyperinsulinemia can cause thyroid tissue proliferation and increase in goiter size [4].

Hypothyroidism in patients with DM is often associated with hypoglycemia and growth disorders in children [2, 14]. Reduction in insulin dosage may be needed to prevent hypoglycemia [2]. SHypo increased the risk of symptomatic hypoglycemia in some studies [15], and in others it had no such effects [14]. SHypo may also be associated with lipid dysmetabolism [14]. Early detection and treatment can reduce the risk of development of hyperlipidemia and atherosclerotic CVD [14]. Hyperthyroidism is less frequent but may be associated with acute diabetic complications and hypertension [14, 16]. The increased metabolism due to hyperthyroidism increases glucose demand and leads to gluconeogenesis and glycogenolysis and may alter blood glucose levels [14].

### 2.2. Cardiometabolic Dysfunction

TH receptors are present on the myocardium and vascular walls. Minor alterations in the circulating levels of TH can affect cardiovascular function, possibly via dyslipidemia, endothelial dysfunction, changes in blood pressure, and direct effects of TH on the myocardium [17].

#### 2.2.1. Direct Effects of Thyroid Hormones on the Myocardium

Both T4 and T3 enter the cardiomyocyte via transporters in the plasma membrane. Inside the cell, T4 is converted to its active form, T3, with the help deiodinase 2 (DIO2). Both T4 and T3 can be inactivated by deiodinase 3 (DIO3). Active T3 binds to TH receptors (TR*α* and TR*β*) in the nucleus and activates genes encoding sodium/potassium-transporting ATPases, myosin heavy chain (MHC)-*α*, and sarcoplasmic/endoplasmic reticulum calcium ATPase 2 and downregulates transcription of MHC-*β* and phospholamban [18].

Inotropic effect of heart is through regulation of gene expression of the *β*1-adrenergic receptors. The chronotropic effect is due to both genomic and nongenomic effects on the adrenergic-receptor complex and on sodium, potassium, and calcium ion channels.

TH also has nongenomic effects on membrane ion channels, mitochondrial membrane, and mitochondriogenesis, and on signaling pathways of cardiomyocytes and vascular smooth muscle cells [18].

In heart diseases, the cellular hypoxia and inflammatory response leads to reduced deiodinase activity, increased DIO3 gene expression, degradation of T3 into inactive metabolites, and reduced availability of T3.

#### 2.2.2. Hyperthyroidism and the Heart

By means of its action on the vascular smooth muscles and endothelial cells, TH increases arterial stiffness in hyperthyroid patients [16]. Hyperthyroidism also increases the resting heart rate, blood volume, stroke volume, myocardial contractility, and ejection fraction [19]. It can cause tachycardia-mediated cardiomyopathy, left ventricular hypertrophy, and up to 300% increase in cardiac output, resembling a state of high adrenergic activity [16]. This leads to high-output cardiac failure, where there is atrial arrhythmia and systolic hypertension with a wide pulse pressure [19].

Chronic subclinical hyperthyroidism alters cardiac morphology and function, enhances systolic function, impairs diastolic function, slows myocardial relaxation, increases left ventricular mass and heart rate, and causes arrhythmias [16]. Endothelial dysfunction and increased thrombogenicity also occur. There is increased risk of hypertension, atrial fibrillation, coronary artery disease (CAD) events, CAD deaths, and total deaths [16, 20].

#### 2.2.3. Hypothyroidism and the Heart

Clinical manifestations of the hemodynamic effects of hypothyroidism are less obvious than those of hyperthyroidism. Masked hypertension, which increases the risk of CVD, is found in about 15% of untreated hypothyroid patients [16]. Reduced heart rate, ventricular filling, and cardiac contractility in hypothyroidism impair diastolic function, decrease cardiac preload, and increase cardiac afterload [16, 20]. The chronotropic and ionotropic functions of the heart are reduced and there is low cardiac output [16, 20]. Heart failure is a rare clinical manifestation, but in nearly one-third of the patients with hypothyroidism, diastolic blood pressure is increased, and the pulse pressure is narrowed [16]. Renin release is decreased, and salt sensitivity is increased; therefore, the blood volume is increased [16]. In severe heart failure patients, euthyroid sick syndrome or low T3 syndrome with normal TSH and T4 levels is common and is associated with poorer outcomes [21]. Atherosclerosis develops in hypothyroidism due to hyperlipidemia, hypercoagulability of blood, endothelial dysfunction, increased arterial stiffness, and hypertension [16]. TH supplementation improves blood lipid profile in overt hypothyroid patients and arterial stiffness in SHypo patients [16].

Compared to euthyroid individuals, patients with SHypo develop hypertension, dyslipidemia, endothelial dysfunction, and myocardial fibrosis more frequently and are more prone to CAD events and CAD deaths [20, 22, 23].

Experimental studies indicate that administration of TH after acute myocardial infarction (AMI) may improve myocardial function by reducing the infarct size [17]. T3 can mitigate hypertension-related vascular dysfunction via its antioxidant effect [16, 24]. However, further research is needed before TH can be therapeutically administered to patients who have had MI or patients with hypertension [18]. SHypo is typically not treated with TH, and treating of low T3 syndrome with TH is currently controversial. However, if abnormal TH levels could be completely reversed, patients could have less severe heart failure [25]. The multicentric ThyroHeart-CHF study is planned to evaluate the safety and efficacy of TH supplementation in patients with chronic heart failure (CHF) complicated with SHypo [26].

#### 2.2.4. Thyroid Dysfunction in Cardiac Disease

In patients with any heart disease, TD can worsen cardiac symptoms or accelerate the underlying cardiac problem.

*(1) Heart Failure.* Both hyperthyroid and hypothyroid states can cause heart failure (HF). Conversely, in patients with established HF, alterations in TH levels can worsen symptoms. Low T3 is common in patients with HF due to impaired T4 to T3 conversion. Low T3 syndrome occurs in 15%–30% of patients with HF and can significantly predict all-cause mortality even after adjustment for ejection fraction and natriuretic peptide levels [27]. After an AMI and in CHF, intracellular and circulating TH levels decrease, and these are associated with poorer outcomes [17]. Treatment of hypothyroidism with TH improves cardiovascular risk factors [17, 27].

*(2) Acute Coronary Syndrome.* Immediately after an AMI, there is a rapid but transient decrease in serum TH levels [27]. Maximal changes occur between 24 and 36 hours of onset of pain [27]. After AMI, low T3 syndrome may occur in nearly 1 in 5 patients, and prevalence of SHypo in nearly 12% of patients [27]. SHypo, SHyper, and low T3 syndrome are associated with a high incidence of cardiac events [27].

*(3) Pulmonary Artery Hypertension and Thyroid Function*. In patients with pulmonary artery hypertension (PAH), thyroid function needs to be evaluated as both hypo- and hyperthyroidism may cause primary PAH [16]. On the other hand, PAH therapy with prostacyclin and its analogs may cause TD [28].

### 2.3. Barometabolic Dysfunctions

#### 2.3.1. Obesity and Thyroid Function

Whether weight changes lead to TD or TD leads to weight changes is controversial. Hypothyroidism is associated with higher body mass index (BMI) and a higher prevalence of obesity [29, 30]. TH plays a significant role in food intake, lipid and glucose metabolism, and fat oxidation. Therefore, irrespective of the physical activity involved, TD affects energy homeostasis and body weight. Even mild TD in the form of SHypo is a risk factor for weight gain and obesity [29]. In hypothyroid patients, minor changes in the dosage of levothyroxine can significantly alter resting energy expenditure (REE) [29]. In obese patients, TH fails to properly activate thermogenesis [31]. Following weight loss, T3 and TSH levels decrease and reduce the REE and total energy expenditure [31]. Weight loss following bariatric surgery promotes decrease in TSH level and leads to greater BMI loss over time [32, 33]. On the other hand, hyperthyroidism is also often associated with weight loss [34]. Patients with higher free tri-iodothyroxine (FT3) show greater weight loss following bariatric surgery [35].

**Figure 1:**
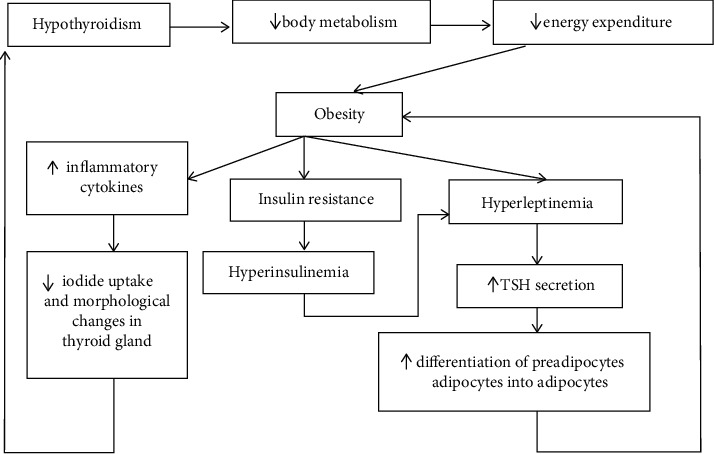
Link between thyroid dysfunction and obesity.

Hyperleptinemia, because of increased adiposity, leads to increased TSH secretion [31]. TSH enhances differentiation of pre-adipocytes into adipocytes [31, 36]. A vicious cycle to hyperleptinemia and hyperthyrotropinemia occurs (Figure 1). Overweight and obese patients often have insulin resistance too, which can stimulate leptin release and lead to hyperleptinemia [31]. The increase in inflammatory cytokines in obesity reduces iodide uptake and may induce morphological changes in the thyroid gland [31].

#### 2.3.2. Dyslipidemia and Thyroid Function

Compared with euthyroid individuals, patients with SHypo have significantly elevated levels of total cholesterol (TC), triglycerides (TG), and low-density lipoprotein-cholesterol (LDL-C) levels and significantly decreased levels of high-density lipoprotein-cholesterol (HDL-C) [37–40]. Such a change is observed with increasing TSH even when TSH is within normal range. This atherogenic dyslipidemia complex and the increased carotid intima-media thickness increase the risk of CVD.

TH affects lipid metabolism by several mechanisms [41]. T3 enhances gene expression of key enzymes involved in lipid metabolism. It induces 3-hydroxy-3-methyl-glutaryl-CoA reductase and increases cholesterol synthesis. It also medicates uptake of cholesterol-rich LDL by directly binding to specific TH-responsive elements on LDL receptors. T3 also protects LDL-C from oxidation.

TH influences HDL-C metabolism by increasing cholesteryl ester transfer protein activity, which transfers cholesterol esters from HDL to triglyceride-rich particles (e.g., LDL and very low-density lipoprotein [VLDL]). TH stimulates lipoprotein lipase and hepatic lipase involved in conversion of intermediate-density lipoproteins to LDL and in turn LDL to small dense LDL (sdLDL). T3 increases levels of apolipoprotein AV, resulting in decreased production, increased lipolysis, and increased hepatic uptake of VLDL-C [41].

### 2.4. Hyperuricemia and Thyroid Function

TH affects uric acid levels by affecting the conversion of purine nucleotides and excretion of uric acid. Increase in uric acid level promotes inflammation and is an important factor leading to dyslipidemia, atherosclerosis, and CVD [42]. Conversely, as TG level increases, the risk for hyperuricemia increases [43].

Early researchers had suggested that hypothyroidism was associated with hyperuricemia, but this has been contradicted by later studies [42]. In hypothyroid patients, TSH seems to be positively correlated with uric acid levels [36, 44, 45]. Some studies have shown a negative correlation, and others have shown no correlation between FT4 and uric acid level [44, 45]. Low uric acid levels can increase the risk of hypertension and high uric acid levels can affect kidney function [42]. In some studies, both hypothyroidism and hyperthyroidism seem to increase the risk for hyperuricemia, and significantly more so among males than among females [46, 47]. In other studies, hypothyroidism and hyperthyroidism were strongly associated with gout but showed weak [48] or no [49] association with hyperuricemia. More studies are warranted to elucidate the influence of gout and hyperuricemia on TD.

### 2.5. Viscerometabolic Dysfunctions

#### 2.5.1. Kidney Disease and Thyroid Function

TH status affects functioning renal mass. Higher TSH levels are also associated with greater risk of developing chronic kidney disease (CKD) [50]. Elderly patients with SHypo and overt hypothyroidism are more likely than euthyroid people to develop CKD [50]. Women are also at a higher risk of CKD [50].

TD is common in patients with nephrotic syndrome (NS) [51]. All patients with nephrotic range proteinuria have increased urinary loss of TH and thyroid-binding globulin [52]. With a normal thyroid reserve, this causes SHypo; with a low thyroid reserve, this leads to overt hypothyroidism [51]. It is essential to recognize and treat this TD with TH replacement. A correlation is also seen between thyroid autoimmunity and NS [52].

#### 2.5.2. Liver Disease and Thyroid Function

TD also alters the liver function. Liver is involved in TH metabolism, transport, and clearance. Changes in TH levels can alter bilirubin metabolism and hepatic circulation.

Though some studies failed to find any association between TD and severity of non-alcoholic fatty liver disease (NAFLD) and non-alcoholic steatohepatitis (NASH), other studies did identify hypothyroidism as an independent risk factor for NAFLD/NASH. A meta-analysis showed that patients with overt hypothyroidism and SHypo are at an increased risk of NAFLD compared to euthyroid subjects [53]. The risk increases significantly with TSH level independent of the TH levels [54]. Hypothyroidism is prevalent in 15.2% to 36.3% of patients with NAFLD [55]. Among patients with NAFLD, those with SHypo or low-normal TH levels have a higher risk of developing advanced fibrosis and NASH [23, 56]. The risk increases significantly with increase in TSH levels.

Insulin resistance, dyslipidemia, obesity, and oxidative stress associated with TD play important roles in the development of NAFLD. TSH itself also has a direct effect on hepatocytes. In hypothyroid patients, there is increased activity of creatinine kinase and lactate dehydrogenase (LDH) [57]. In hypothyroid patients, a positive correlation has been found between TSH levels and levels of alanine aminotransferase, aspartate aminotransferase, and albumin [45]. Cirrhotic patients have high serum bilirubin, prothrombin time, international normalized ratio, and TSH levels and low serum albumin, T3, FT3, and FT4 levels [58, 59]. As TH levels correlate with severity of the disease, they can be used as a prognostic marker for liver cirrhosis [58, 59]. Studies have shown that 3,5-diiodo-l-thyronine (T2), a TH derivative [53], and levothyroxine [60] improved lipid metabolism and are protective against NAFLD.

**Figure 2:**
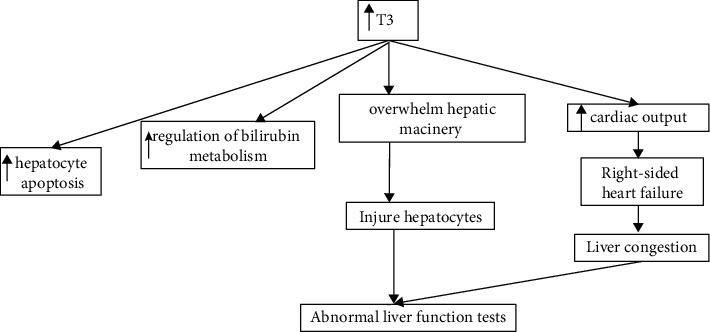
Link between hyperthyroidism and abnormal liver function.

Hyperthyroidism also alters liver function by several different mechanisms (Figure 2).

Patients with acute thyrotoxicosis may show liver dysfunction ranging from mild transaminitis to profound cholestatic liver dysfunction [61, 62].

#### 2.5.3. Polycystic Ovary Syndrome and Thyroid Function

TD is also closely linked to polycystic ovary syndrome (PCOS). Though the causality is uncertain, both TD and PCOS have common risk factors and pathophysiological abnormalities. Hypothyroidism must be excluded before diagnosing PCOS. Increased BMI, insulin resistance, hyperleptinemia, and signs of deranged autoimmunity are common to both [63]. In hypothyroidism, prolactin and TSH levels rise [63]. Prolactin affects the ratio of luteinizing hormone and follicle stimulating hormone. It also increases the level of dehydroepiandrosterone. These fluctuating hormone levels inhibit ovulation, increase ovarian volume, and lead to cysts. They possibly lead to collagen deposits in the ovaries too. Long-standing, severe primary hypothyroidism can change ovarian morphology to an extent that may be confused with ovarian malignancies [63]. While hypothyroidism can lead to polycystic morphological changes in the ovaries, its causality to PCOS has not been established [63].

AITD has been observed in 18%–40% of women with PCOS [64]. Incidence of SHypo, goiters, and thyroid autoimmunity is higher among females with PCOS, and they have higher anti-TPO levels, larger thyroid volumes, and more hypoechogenic thyroids than those without [63, 65, 66]. In euthyroid patients with PCOS, higher TSH is associated with increased prevalence of hyperandrogenic phenotype [67]. A recently published Korean study reported that AITD is not more prevalent among women with PCOS but when women have PCOS and AITD, they are more likely to show higher adiposity and insulin resistance [68].

## 3. Drugs Altering Metabolic and Thyroid Functions

Table 1 summarizes the common drugs used in the various metabolic conditions discussed thus far and their impact on thyroid function.

### 3.1. Medications for Glucometabolic Dysfunction

Metformin is beneficial in both T2DM and TD. In addition to providing glycemic control in T2DM, it reduces the TSH levels in TD. However, other antidiabetic drugs such as older sulfonylureas, pioglitazone, and thiazolidinediones can adversely affect TD (Table 1). Antithyroid drugs such as methimazole can worsen glycemic control in T2DM patients [4]. Glucocorticoids used in the treatment of thyrotoxicosis can cause hyperglycemia [69]. Methimazole-induced insulin autoimmune syndrome resulting in hypoglycemia has also been reported [70].

### 3.2. Medications for Cardiometabolic Dysfunction

The high concentration of iodine in cardiac medication amiodarone may induce TD [20]. Several antihypertensive drugs may affect TH concentrations. Beta-blockers block conversion of T4 to T3; prazosin decreases T3 and increases T4 and TSH levels. Propranolol, digoxin, and oral anticoagulants used for CVD can alter TH levels and must be used cautiously [71].

### 3.3. Medications for Barometabolic Disorders

Treatment of SHypo decreases the risk of dyslipidemia and CVD [16]. Medications used to treat dyslipidemia, such as cholestyramine and colestipol, can alter TH level in patients with TD [71]. Effects of statins are considerably lower in patients with TD than in euthyroid individuals. Ezetimibe, in addition to inhibiting synthesis and intestinal absorption of TC, enhances conversion of T4 to T3 (Table 1; [72]) Selective TH mimetics, such as eprotirome, lower LDL-C levels when added to statins but cause hepatic injury [73].

### 3.4. Medications for Viscerometabolic Disorders

TH replacement therapy in SHypo patients with CKD helps preserve renal function [74]. TH therapy is also an independent predictor of renal outcome in these patients. Medications, such as methimazole and propylthiouracil, used to treat hyperthyroidism, can also alter liver function [61, 75]. T4 supplementation has benefits on NAFLD in patients with significant SHypo or SHypo with dyslipidemia [76].

## 4. Pragmatic Thyrovigilance

Table 2 summarizes the metabolic conditions that need pragmatic thyrovigilance.

### 4.1. Diabetes

International guidelines provide clear recommendations for thyrovigilance in patients with T1DM [5, 77–80]; however, for T2DM patients, some guidelines lack recommendations on monitoring of thyroid functions [5, 77–79], whereas others recommend thyroid function test (TFT) at baseline but do not advocate routine annual thyroid monitoring [80].

Kadiyala et al. [81] suggested screening of all diabetes patients for TSH and anti-TPO at baseline. In euthyroid T1DM, annual TSH screening is recommended for all while, in euthyroid T2DM, it is needed only when TSH level is ≥ 2.0 mU/L or anti-TPO is detectable. In others, TSH screening is advised every 3–5 years. Thus, a systematic approach for thyroid testing in patients with T1DM and T2DM is needed.

### 4.2. Heart Disease

The European Society of Cardiology (ESC) recommends that patients suspected with CAD must be examined for comorbid thyroid disease and if there is clinical suspicion of TD, blood TH levels must be measured [82]. ESC also recommends standard thyroid function test during initial evaluation for supraventricular tachycardia [62] and heart failure [83]. The American College of Cardiology also recommends TSH as a basic test for primary hypertension evaluation [84].

Real-world evidence suggests that timely and adequate treatment of TD with careful monitoring can reduce the risk of CVD [85]. Delayed recognition of subclinical TD has unfavorable cardiovascular effects. Hence, careful thyrovigilance may be necessary for those with other risks for CVD. TH replacement therapy should be initiated cautiously in patients with CAD to avoid exacerbation of angina pectoris or precipitation of AMI, ventricular arrhythmias, or congestive heart failure [86].

### 4.3. Obesity and Dyslipidemia

The present European Society of Endocrinology Clinical Guideline on the Endocrine Work-up in Obesity acknowledges the high prevalence of hypothyroidism in obesity and recommends thyroid function screening for all patients with obesity [87]. Although biochemical screening for TD is recommended for all patients with dyslipidemia [78], a majority of newly diagnosed hyperlipidemic patients are not screened for TD [88].

### 4.4. Hyperuricemia

Monitoring of uric acid levels in TD may be needed as TH replacement therapy can improve insulin sensitivity in SHypo patients and modulate the uric acid levels [89].

### 4.5. Kidney and Liver Diseases

Both overtly hypothyroid and SHypo patients may have high serum urea and creatinine levels, and regular monitoring of renal function is suggested as the renal dysfunction in TD is often reversible [44, 90]. Resolution of hypothyroidism is associated with significant reduction in serum creatinine levels [91]. As both hypothyroidism and NS have similar clinical presentations such as facial puffiness, weight gain, and fatigue, TD can be easily missed before it becomes severe enough to cause impairments in other major organs of the body [52]. If left untreated, it can impair the renal function further. Physicians need to be vigilant about TD when managing NS patients.

Liver function tests are recommended in patients with TD, and thyrovigilance is recommended in those with altered liver function.

### 4.6. Polycystic Ovarian Syndrome

Even in the absence of overt symptoms suggesting TD, screening for TH and thyroid-specific autoantibodies should be considered in women with PCOS [64]. Women who have difficulty in getting pregnant should be screened for both TD and PCOS.

## 5. Summary

An increased prevalence of TD is seen in patients with DM, and TD is a risk factor for new onset DM especially in those with prediabetes. Both DM and TD increase the risk for CVD. Even subclinical TD can increase the risk of diabetic complications. Comorbid TD can worsen glycemic control while comorbid DM can worsen thyroid function. Antidiabetics can alter thyroid function, and antithyroid drugs can worsen DM. Though, for patients with T1DM patients, international guidelines recommend thyroid screening via blood tests at baseline and periodically thereafter, for T2DM, there are no specific recommendations.

Both hypothyroidism and hyperthyroidism increase the risk of CVD. Overt as well as subclinical hyperthyroidism increases the risk of hypertension, heart failure, atrial fibrillation, CAD events, CAD deaths, and total deaths. Treatment of hypothyroidism with TH supplementation improves the risk for CVD. Treatment of SHypo with TH supplementation is controversial but some data suggest that severity of heart failure can be reduced if the abnormal TH levels can be completely reversed.

Even mild asymptomatic TD increases the risk for weight gain and obesity. Overweight and obese individuals are at higher risk of TD especially those with SHypo, which may be an effect of overweight per se. TSH levels improve with weight loss as can be seen in subjects undergoing bariatric surgery. Hyperthyroidism is also associated with weight loss. Recent guidelines recommend screening of all obese patients for TD.

Current guidelines recommend against screening of TD in asymptomatic, non-pregnant adults in primary care [92, 93]. This is based on insufficient evidence supporting clinical benefit of treating asymptomatic, screen-detected hypothyroidism. However, clinicians should remain alert to signs and symptoms of TD, maintain high index of clinical suspicion, and investigate accordingly [87]. This vigilance is of particular importance in disorders of glucose, lipid, and protein metabolism as seen in the case of diabetes, obesity, dyslipidemia, hyperuricemia, and organ-specific disorders especially those of the liver, kidney, and the ovaries. Drug-induced TD should be considered when TH levels are inconsistent with clinical findings or when patients are on medications that can alter thyroid function.

## Figures and Tables

**Table 1:** Common drugs used in metabolic disorders that can alter thyroid function tests and should be used with caution.

Metabolic disorder	Drug	Effect
Glucometabolic dysfunction	Metformin	↓ TSH levels in hypothyroid patients
	Pioglitazone	↓ TSH levels in hypothyroid patients
	Thiazolidinediones	Can induce thyroid-associated orbitopathy
Cardiometabolic dysfunction	Amiodarone	Can cause both hypothyroidism and thyrotoxicosis
	Beta blockers	↓ circulating T3 levels
	Prazosin	↑ TSH, ↑ T4, ↓ T3
	Sodium nitroprusside	Prevents hypothyroidism in patients undergoing coronary artery bypass grafting
Barometabolic disorders	Cholestyramine	↓TH levels; can impair levothyroxine absorption
	Colestipol	Transiently ↓T3 levels in some patients
	Statins	May have antiproliferative effect on thyroid cells; can alter thyroid size; effect of statin is lowered in TD
	Ezetimibe	Enhance conversion of T4 to T3
Viscerometabolic disorders	Interferon therapy used in liver and kidney diseases	Thyroid inflammation (both autoimmune and non-autoimmune), and either hyper- or hypothyroidism, which improve following discontinuation of interferon therapy
	Alemtuzumab used as induction agent in renal transplantation	Autoimmune Graves' disease
	Lenalidomide used in renal cancer therapy	Hypothyroidism or hyperthyroidism
	Sunitinib used in renal cancer therapy	Transient hypothyroidism
	Clomiphene citrate used in PCOS	Associated with increased risk of thyroid cancer

PCOS, polycystic ovary syndrome; T3, triiodothyronine; T4, thyroxine; TD, thyroid dysfunction; TH, thyroid hormone; TSH, thyroid stimulating hormone.

**Table 2:** Common dysmetabolic states where pragmatic thyrovigilance is required.

Class	Condition
Glucometabolic disorders	Type 1 diabetes mellitus
	Type 2 diabetes mellitus
Cardiometabolic disorders	Hypertension
	Heart failure
	Acute coronary syndrome
Barometabolic disorders	Obesity
	Hyperlipidemia
	Hyperuricemia
Viscerometabolic disorders	Kidney dysfunction
	Liver dysfunction
	Polycystic ovary disorder

## Data Availability

As this is a review article, no original data are applicable.

## References

[1] Rugge B., Balshem H., Sehgal R., Relevo R., Gorman P., Helfand M. (2011). *Introduction. Screen Treat Subclin Hypothyroidism Hyperthyroidism*.

[2] Biondi B., Kahaly G. J., Robertson R. P. (2019). Thyroid dysfunction and diabetes mellitus: two closely associated disorders. *Endocrine Reviews*.

[3] Chaker L., Ligthart S., Korevaar T. I. M. (2016). Thyroid function and risk of type 2 diabetes: a population-based prospective cohort study. *BMC Medicine*.

[4] Kalra S., Aggarwal S., Khandelwal D. (2019). Thyroid dysfunction and type 2 diabetes mellitus: screening strategies and implications for management. *Diabetes Therapy*.

[5] American Diabetes Association (2020). Comprehensive medical evaluation and assessment of comorbidities: standards of medical care in diabetes—2020. *Diabetes Care*.

[6] Shun C. B., Donaghue K. C., Phelan H., Twigg S. M., Craig M. E. (2014). Thyroid autoimmunity in type 1 diabetes: systematic review and meta-analysis. *Diabetic Medicine*.

[7] Libman I. M., Sun K., Foley T. P., Becker D. J. (2008). Thyroid autoimmunity in children with features of both type 1 and type 2 diabetes. *Pediatric Diabetes*.

[8] Fatourechi A., Ardakani H. M., Sayarifard F., Sheikh M. (2017). Hypothyroidism among pediatric patients with type 1 diabetes mellitus, from patients’ characteristics to disease severity. *Clinical Pediatric Endocrinology*.

[9] Al-Geffari M., Ahmad N. A., Al-Sharqawi A. H., Youssef A. M., Alnaqeb D., Al-Rubeaan K. (2013). Risk factors for thyroid dysfunction among type 2 diabetic patients in a highly diabetes mellitus prevalent society. *International Journal of Endocrinology*.

[10] Díez J. J., Iglesias P. (2012). An analysis of the relative risk for hypothyroidism in patients with type 2 diabetes. *Diabetic Medicine*.

[11] Han C., He X., Xia X. (2015). Subclinical hypothyroidism and type 2 diabetes: a systematic review and meta-analysis. *PLoS One*.

[12] Gronich N., Deftereos S. N., Lavi I., Persidis A. S., Abernethy D. R., Rennert G. (2015). Hypothyroidism is a risk factor for new-onset diabetes: a cohort study. *Diabetes Care*.

[13] Cho J. H., Kim H. J., Lee J. H. (2016). Poor glycemic control is associated with the risk of subclinical hypothyroidism in patients with type 2 diabetes mellitus. *The Korean Journal of Internal Medicine*.

[14] Krzewska A., Ben-Skowronek I. (2016). Effect of associated autoimmune diseases on type 1 diabetes mellitus incidence and metabolic control in children and adolescents. *BioMed Research International*.

[15] Mohn A., Di Michele S., Di Luzio R., Tumini S., Chiarelli F. (2002). The effect of subclinical hypothyroidism on metabolic control in children and adolescents with type 1 diabetes mellitus. *Diabetic Medicine*.

[16] Berta E., Lengyel I., Halmi S. (2019). Hypertension in thyroid disorders. *Frontiers in Endocrinology*.

[17] Jabbar A., Pingitore A., Pearce S. H. S., Zaman A., Iervasi G., Razvi S. (2017). Thyroid hormones and cardiovascular disease. *Nature Reviews Cardiology*.

[18] Razvi S. (2019). Novel uses of thyroid hormones in cardiovascular conditions. *Endocrine*.

[19] Osuna P. M., Udovcic M., Sharma M. D. (2017). Hyperthyroidism and the heart. *Methodist DeBakey Cardiovascular Journal*.

[20] Vargas-Uricoechea H., Bonelo-Perdomo A., Sierra-Torres C. H. (2014). Effects of thyroid hormones on the heart. *Clínica e Investigación en Arteriosclerosis*.

[21] Fraczek-Jucha M., Zbierska-Rubinkiewicz K., Kabat M. (2019). Low triiodothyronine syndrome and selenium deficiency-undervalued players in advanced heart failure? A single center pilot study. *BMC Cardiovascular Disorders*.

[22] Gong N., Gao C., Chen X., Fang Y., Tian L. (2019). Endothelial function in patients with subclinical hypothyroidism: a meta-analysis. *Hormone and Metabolic Research*.

[23] Bano A., Chaker L., Muka T. (2020). Thyroid function and the risk of fibrosis of the liver, heart and lung in humans: a systematic review and meta-analysis. *Thyroid: Official Journal of the American Thyroid Association*.

[24] Carrillo-Sepulveda M. A., Panackal A., Maracheril R. (2019). Triiodothyronine reduces vascular dysfunction associated with hypertension by attenuating protein kinase g/vasodilator-stimulated phosphoprotein signaling. *Journal of Pharmacology and Experimental Therapeutics*.

[25] Cappola A. R., Desai A. S., Medici M. (2019). Thyroid and cardiovascular disease. *Circulation*.

[26] Zhang X., Wang W., Zhang K. (2019). Efficacy and safety of levothyroxine (L-T4) replacement on the exercise capability in chronic systolic heart failure patients with subclinical hypothyroidism: study protocol for a multi-center, open label, randomized, parallel group trial (ThyroHeart-CHF). *Trials*.

[27] Razvi S., Jabbar A., Pingitore A. (2018). Thyroid hormones and cardiovascular function and diseases. *Journal of the American College of Cardiology*.

[28] Menon A. A., Sahay S., Braverman L. E., Farber H. W. (2019). Thyroid dysfunction in patients with pulmonary artery hypertension (PAH): the effect of therapies affecting the prostanoid pathway. *Lung*.

[29] Sanyal D., Raychaudhuri M. (2016). Hypothyroidism and obesity: an intriguing link. *Indian Journal of Endocrinology and Metabolism*.

[30] Martínez Escudé A., Pera G., Arteaga I. (2020). Relationship between hypothyroidism and non-alcoholic fatty liver disease in the Spanish population. *Medicina Clínica (English Edition)*.

[31] Yadav J., Jain N., Dayal D. (2018). Alterations of thyroid function in overweight and obese children: an update. *Indian Journal of Child Health*.

[32] Neves J. S., Castro Oliveira S., Castro Oliveira S. (2018). Effect of weight loss after bariatric surgery on thyroid-stimulating hormone levels in patients with morbid obesity and normal thyroid function. *Obesity Surgery*.

[33] Juiz-Valiña P., Outeiriño-Blanco E., Pértega S. (2019). Effect of weight loss after bariatric surgery on thyroid-stimulating hormone levels in euthyroid patients with morbid obesity. *Nutrients*.

[34] Kyriacou A., Kyriacou A., Makris K. C., Syed A. A., Perros P. (2019). Weight gain following treatment of hyperthyroidism-a forgotten tale. *Clinical Obesity*.

[35] Neves J. S., Souteiro P., Souteiro P. (2019). Preoperative thyroid function and weight loss after bariatric surgery. *International Journal of Obesity*.

[36] Chang Y.-C., Hua S.-C., Chang C.-H. (2019). High TSH level within normal range is associated with obesity, dyslipidemia, hypertension, inflammation, hypercoagulability, and the metabolic syndrome: a novel cardiometabolic marker. *Journal of Clinical Medicine*.

[37] Rastgooye Haghi A., Solhjoo M., Tavakoli M. H. (2017). Correlation between subclinical hypothyroidism and dyslipidemia. *Iranian Journal of Pathology*.

[38] Unal E., Akın A., Yıldırım R., Demir V., Yildiz İ., Haspolat Y. K. (2017). Association of subclinical hypothyroidism with dyslipidemia and increased carotid intima-media thickness in children. *Journal of Clinical Research in Pediatric Endocrinology*.

[39] Hussain A., Elmahdawi A. M., Elzeraidi N. E.-H., Nouh F., Algathafi K. (2020). The effects of dyslipidemia in subclinical hypothyroidism. *Cureus*.

[40] Dey A., Kanneganti V., Das D. (2019). A study of the cardiac risk factors emerging out of subclinical hypothyroidism. *Journal of Family Medicine and Primary Care*.

[41] Rizos C. V., Elisaf M. S., Liberopoulos E. N. (2011). Effects of thyroid dysfunction on lipid profile. *The Open Cardiovascular Medicine Journal*.

[42] Chao G., Zhu Y., Fang L. (2019). Retrospective analysis of the correlation between uric acid and thyroid hormone in people with normal thyroid function. *Journal of Diabetes Research*.

[43] Hou Y.-L., Yang X.-L., Wang C.-X., Zhi L.-X., Yang M.-J., You C.-G. (2019). Hypertriglyceridemia and hyperuricemia: a retrospective study of urban residents. *Lipids in Health and Disease*.

[44] Saini V., Yadav A., Arora M. K., Arora S., Singh R., Bhattacharjee J. (2012). Correlation of creatinine with TSH levels in overt hypothyroidism-a requirement for monitoring of renal function in hypothyroid patients?. *Clinical Biochemistry*.

[45] Arora S., Chawla R., Tayal D., Gupta V. K., Sohi J. S., Mallika V. (2009). Biochemical markers of liver and kidney function are influenced by thyroid function-a case-controlled follow up study in Indian hypothyroid subjects. *Indian Journal of Clinical Biochemistry*.

[46] Zhang J., Meng Z., Zhang Q. (2016). Gender impact on the correlations between subclinical thyroid dysfunction and hyperuricemia in Chinese. *Clinical Rheumatology*.

[47] Liu X., Zhang J., Meng Z. (2019). Gender impact on the correlations between Graves’ hyperthyroidism and hyperuricemia in Chinese. *Irish Journal of Medical Science (1971)*.

[48] See L.-C., Kuo C.-F., Yu K.-H. (2014). Hyperthyroid and hypothyroid status was strongly associated with gout and weakly associated with hyperuricaemia. *PLoS One*.

[49] Xu J., Wang B., Li Q. (2019). Risk of thyroid disorders in patients with gout and hyperuricemia. *Hormone and Metabolic Research*.

[50] Chuang M.-H., Liao K.-M., Hung Y.-M., Wang P. Y.-P., Chou Y.-C., Chou P. (2016). Abnormal thyroid-stimulating hormone and chronic kidney disease in elderly adults in Taipei city. *Journal of the American Geriatrics Society*.

[51] Mario F. D., Pofi R., Gigante A. (2017). Hypothyroidism and nephrotic syndrome: why, when and how to treat. *Current Vascular Pharmacology*.

[52] Jain D., Aggarwal H. K., Pavan Kumar Y. M., Jain P. (2019). Evaluation of thyroid dysfunction in patients with nephrotic syndrome. *Medicine and Pharmacy Reports*.

[53] He W., An X., Li L. (2017). Relationship between hypothyroidism and non-alcoholic fatty liver disease: a systematic review and meta-analysis. *Frontiers in Endocrinology*.

[54] Guo Z., Li M., Han B., Qi X. (2018). Association of non-alcoholic fatty liver disease with thyroid function: a systematic review and meta-analysis. *Digestive and Liver Disease*.

[55] Eshraghian A., Hamidian Jahromi A. (2014). Non-alcoholic fatty liver disease and thyroid dysfunction: a systematic review. *World Journal of Gastroenterology*.

[56] Kim D., Kim W., Joo S. K., Bae J. M., Kim J. H., Ahmed A. (2018). Subclinical hypothyroidism and low-normal thyroid function are associated with nonalcoholic steatohepatitis and fibrosis. *Clinical Gastroenterology and Hepatology*.

[57] McGrowder D., Gordon L., Rawlins J., Fraser Y., Crawford T. (2011). Serum creatine kinase and lactate dehydrogenase activities in patients with thyroid disorders. *Nigerian Journal of Clinical Practice*.

[58] Punekar P., Sharma A. K., Jain A. (2018). A study of thyroid dysfunction in cirrhosis of liver and correlation with severity of liver disease. *Indian Journal of Endocrinology and Metabolism*.

[59] Patira N. K., Salgiya N., Agrawal D. (2019). Correlation of thyroid function test with severity of liver dysfunction in cirrhosis of liver. *The Journal of the Association of Physicians of India*.

[60] Chi H.-C., Tsai C.-Y., Tsai M.-M., Yeh C.-T., Lin K.-H. (2019). Molecular functions and clinical impact of thyroid hormone-triggered autophagy in liver-related diseases. *Journal of Biomedical Science*.

[61] Fatima S., Puri R., Patnaik S., Mora J. (2016). When a toxic thyroid makes the liver toxic: a case of thyroid storm complicated by acute liver failure. *AACE Clinical Case Reports*.

[62] Brugada J., Katritsis D. G., Arbelo E. (2020). 2019 ESC guidelines for the management of patients with supraventricular tachycardia the task force for the management of patients with supraventricular tachycardia of the European society of Cardiology (ESC) developed in collaboration with the association for European paediatric and congenital Cardiology (AEPC). *European Heart Journal*.

[63] Singla R., Gupta Y., Khemani M., Aggarwal S. (2015). Thyroid disorders and polycystic ovary syndrome: an emerging relationship. *Indian Journal of Endocrinology and Metabolism*.

[64] Romitti M., Fabris V. C., Ziegelmann P. K., Maia A. L., Spritzer P. M. (2018). Association between PCOS and autoimmune thyroid disease: a systematic review and meta-analysis. *Endocrine Connections*.

[65] Yu Q., Wang J.-B. (2016). Subclinical hypothyroidism in PCOS: impact on presentation, insulin resistance, and cardiovascular risk. *BioMed Research International*.

[66] Ding X., Yang L., Wang J. (2018). Subclinical hypothyroidism in polycystic ovary syndrome: a systematic review and meta-analysis. *Frontiers in Endocrinology*.

[67] Cai J., Zhang Y., Wang Y. (2019). High thyroid stimulating hormone level is associated with hyperandrogenism in euthyroid polycystic ovary syndrome (PCOS) women, independent of age, BMI, and thyroid autoimmunity: a cross-sectional analysis. *Frontiers in Endocrinology*.

[68] Kim J. J., Yoon J. W., Kim M. J., Kim S. M., Hwang K. R., Choi Y. M. (2020). Thyroid autoimmunity markers in women with polycystic ovary syndrome and controls. *Human Fertility*.

[69] Kalra S., Khandelwal D. (2018). Thyrovigilance in diabetes; glucovigilance in thyroidology. *The Journal of the Pakistan Medical Association*.

[70] Jain N., Savani M., Agarwal M., Kadaria D. (2016). Methimazole-induced insulin autoimmune syndrome. *Therapeutic Advances in Endocrinology and Metabolism*.

[71] Kavanagh S., Boparai P. (2015). Thyroid dysfunction and drug interactions. *The Pharmaceutical Journal*.

[72] Duntas L. H., Brenta G. (2018). A renewed focus on the association between thyroid hormones and lipid metabolism. *Frontiers in Endocrinology*.

[73] Sjouke B., Langslet G., Ceska R. (2014). Eprotirome in patients with familial hypercholesterolaemia (the AKKA trial): a randomised, double-blind, placebo-controlled phase 3 study. *The Lancet Diabetes & Endocrinology*.

[74] Shin D. H., Lee M. J., Kim S. J. (2012). Preservation of renal function by thyroid hormone replacement therapy in chronic kidney disease patients with subclinical hypothyroidism. *The Journal of Clinical Endocrinology & Metabolism*.

[75] Tufton N., Hashim N., Sze C., Waterhouse M. (2015). A case of thyroid storm complicated by acute hepatitis due to propylthiouracil treatment. *Endocrinology, Diabetes & Metabolism Case Reports*.

[76] Liu L., Yu Y., Zhao M. (2017). Benefits of levothyroxine replacement therapy on nonalcoholic fatty liver disease in subclinical hypothyroidism patients. *International Journal of Endocrinology*.

[77] Alexander E. K., Pearce E. N., Brent G. A. (2017). 2017 guidelines of the American thyroid association for the diagnosis and management of thyroid disease during pregnancy and the postpartum. *Thyroid*.

[78] Garber J. R., Cobin R. H., Gharib H. (2012). Clinical practice guidelines for hypothyroidism in adults: cosponsored by the American Association of clinical endocrinologists and the American thyroid association. *Endocrine Practice*.

[79] Mahmud F. H., Elbarbary N. S., Fröhlich-Reiterer E. (2018). ISPAD Clinical Practice Consensus Guidelines 2018: other complications and associated conditions in children and adolescents with type 1 diabetes. *Pediatric Diabetes*.

[80] British Thyroid Association (2006). *The Association for Clinical Biochemistry*.

[81] Kadiyala R., Peter R., Okosieme O. E. (2010). Thyroid dysfunction in patients with diabetes: clinical implications and screening strategies. *International Journal of Clinical Practice*.

[82] Knuuti J., Wijns W., Saraste A. (2020). 2019 ESC Guidelines for the diagnosis and management of chronic coronary syndromes. The Task Force for the diagnosis and management of chronic coronary syndromes of the European Society of Cardiology (ESC). *European Heart Journal*.

[83] Ponikowski P., Voors A. A., Anker S. D. (2016). 2016 ESC Guidelines for the diagnosis and treatment of acute and chronic heart failure. *European Heart Journal*.

[84] Whelton P. K., Carey R. M., Aronow W. S. (2018). 2017 ACC/AHA/AAPA/ABC/ACPM/AGS/APhA/ASH/ASPC/NMA/PCNA guideline for the prevention, detection, evaluation, and management of high blood pressure in adults: a report of the American College of Cardiology/American Heart Association task force on clinical practice guidelines. *Journal of the American College of Cardiology*.

[85] Lillevang-Johansen M., Abrahamsen B., Jørgensen H. L., Brix T. H., Hegedüs L. (2019). Duration of hyperthyroidism and lack of sufficient treatment are associated with increased cardiovascular risk. *Thyroid*.

[86] Khandelwal D., Tandon N. (2012). Overt and subclinical hypothyroidism. *Drugs*.

[87] Pasquali R., Casanueva F., Haluzik M. (2020). European society of endocrinology clinical practice guideline: endocrine work-up in obesity. *European Journal of Endocrinology*.

[88] Willard D. L., Leung A. M., Pearce E. N. (2014). Thyroid function testing in patients with newly diagnosed hyperlipidemia. *JAMA Internal Medicine*.

[89] Desideri G., Bocale R., D’Amore A. M. (2019). Thyroid hormones modulate uric acid metabolism in patients with recent onset subclinical hypothyroidism by improving insulin sensitivity. *Internal and Emergency Medicine*.

[90] Sayari S., Molaei Z., Torabi Z. (2018). The relationship between subclinical hypothyroidism and serum levels of uric acid and creatinine in children aged 2–14 years. *Annals of Pediatric Endocrinology & Metabolism*.

[91] Dutta D., Garg A., Khandelwal D., Kalra S., Mittal S., Chittawar S. (2019). Thyroid symptomatology across the spectrum of hypothyroidism and impact of levothyroxine supplementation in patients with severe primary hypothyroidism. *Indian Journal of Endocrinology and Metabolism*.

[92] LeFevre M. L., U.S. Preventive Services Task Force (2015). Screening for thyroid dysfunction: U.S. Preventive Services Task Force recommendation statement. *Annals of Internal Medicine*.

[93] Birtwhistle R., Morissette K., Dickinson J. A. (2019). Recommendation on screening adults for asymptomatic thyroid dysfunction in primary care. *Canadian Medical Association Journal*.

